# Third Course of Lectures

**Published:** 1856-10

**Authors:** 


### EDITORIAL AND MISCELLANEOUS.

With this No. of our Journal we send out the announcement of our third course of Lectures. We are satisfied that the accompanying Catalogue of Students and graduates of the last course shows an increase upon the first sufficiently large to warrant us in the conclusion that the Atlanta Medical College will very soon number a class equal, with very few exceptions, to any school in the Union. This is said in no boasting spirit. The Faculty, it is true, have endeavored to discharge their duties as teachers—others have done the same; and we are glad to see all institutions prosper that afford the proper amount of medical instruction. We hope no jealousy toward sister institutions will ever be indulged by any member of the Faculty here. If our position and facilities for instruction, make it to the interest of the student to take a course of lectures in Atlanta, we expect them to do so, not otherwise. If we fall short of other colleges in the curriculum of studies, or in affording the proper means of rapid advancement, we do not ask, we do not expect, we do not desire the medical student to waste his time and money with us. If we fall below the standard of requirements adopted by other institutions, we could not expect their confidence and respect, nor the patronage of intelligent students of medicine.

The College building, which for want of means has progressed tardily, has now its walls complete; and with the fulfillment of contracts already made for other work, which will be rapidly carried on, ample lecture rooms, dissecting room, museum, &c., in a substantial stone building, will be afforded. Though the rooms occupied heretofore by the kindness of our city authorities, were spacious and comfortable, the want of laboratory, and permanent and suitable dissecting rooms was felt by those requiring such apartments during the College exercises. As the announcement makes known, all things will be in readiness for the next course.

